# Targeted expression of *Vitreoscilla* hemoglobin improves the production of tropane alkaloids in *Hyoscyamus niger* hairy roots

**DOI:** 10.1038/s41598-018-36156-y

**Published:** 2018-12-19

**Authors:** Zhiying Guo, Hexin Tan, Zongyou Lv, Qian Ji, Yuxiang Huang, Jingjing Liu, Donghong Chen, Yong Diao, Jinping Si, Lei Zhang

**Affiliations:** 1 School of Medicine, School of Biomedical Science, Huaqiao University, Quanzhou, Fujian, 362021 China; 2 State Key Laboratory of Subtropical Silviculture, Zhejiang A & F University, Hangzhou, Zhejiang, 311300 China; 3 Department of Pharmaceutical Botany, School of Pharmacy, Naval Medical University (Second Military Medical University), Shanghai, 200433 China

## Abstract

Under hypoxic conditions, the expression of *Vitreoscilla* hemoglobin (VHb) in plants is proposed to increase the productivity of certain oxygen-requiring metabolic pathways by promoting the delivery of oxygen. Tropane alkaloids (TAs) are a class of important plant secondary metabolites with significant medicinal value; the final step in their biosynthesis requires oxygen. Whether heterologous expression of VHb, especially in different subcellular compartments, can accelerate the accumulation of TAs is not known. Herein, the effect of heterologous expression of VHb in different subcellular locations on the TA profile of *H*. *niger* hairy roots was investigated. The targeted expression of VHb in the plastids (using *p*VHb-RecA construct), led to the accumulation of 197.68 μg/g hyoscyamine in the transgenic *H*. *niger* hairy roots, which was 1.25-fold of the content present in the lines in which VHb expression was not targeted, and 3.66-fold of that present in the wild type (WT) lines. The content of scopolamine was increased by 2.20- and 4.70-fold in the *p*VHb-RecA transgenic lines compared to that in the VHb transgenic and WT lines. Our results demonstrate that VHb could stimulate the accumulation of TAs in the transgenic *H*. *niger* hairy roots. Quantitative RT-PCR analysis revealed that the expression of key genes involved in TA biosynthesis increased significantly in the VHb transgenic lines. We present the first description of a highly efficient strategy to increase TA content in *H*. *niger*. Moreover, our results also shed light on how the production of desired metabolites can be efficiently enhanced by using more accurate and appropriate genetic engineering strategies.

## Introduction

**Figure 1:**
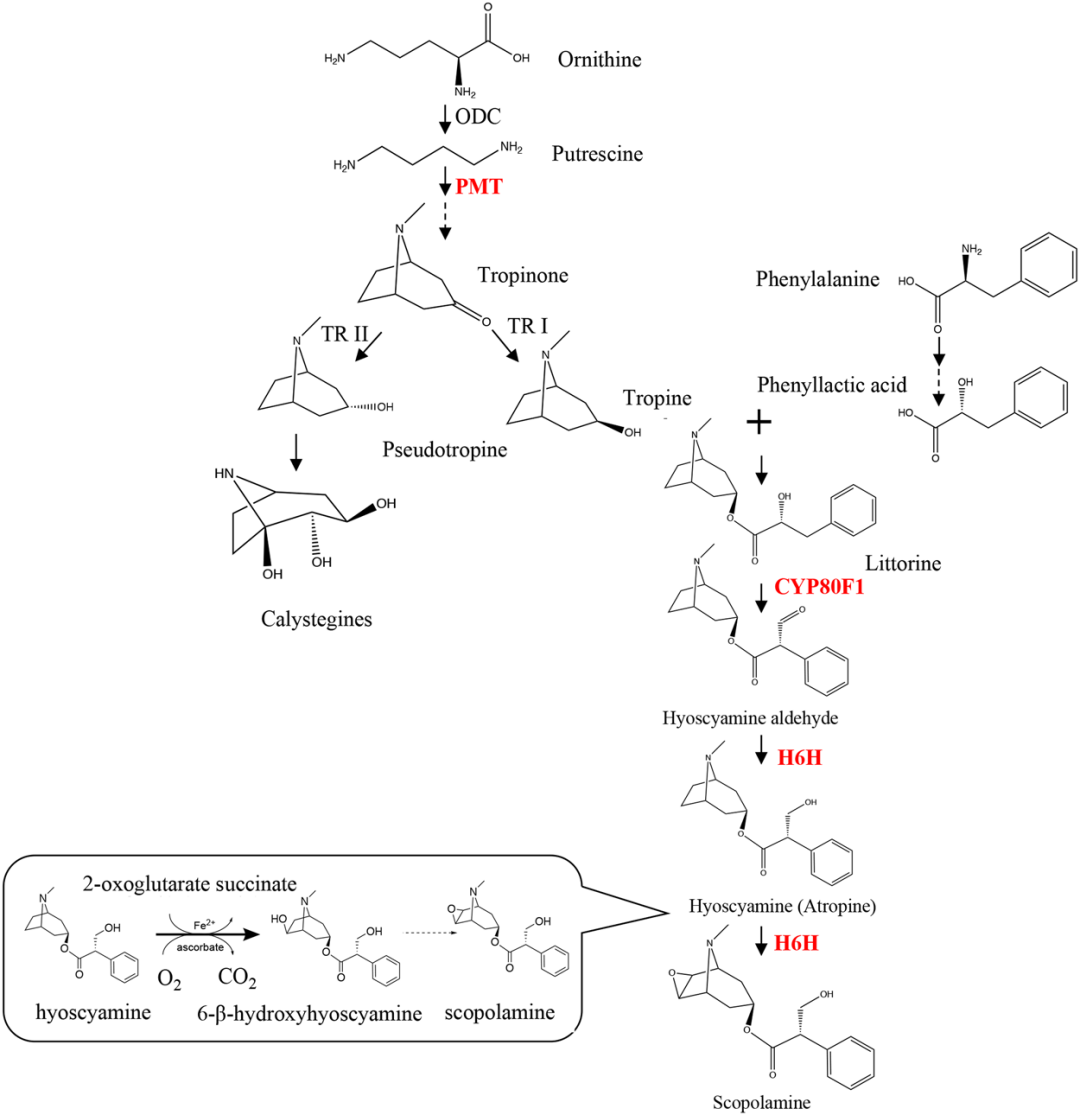
Biosynthetic pathway of tropane alkaloids. The abbreviations used for the names of enzymes are as follows: PMT, putrescine-*N*-methyltransferase; TR I, tropinone reductase I; TR II, tropinone reductase II; CYP80F1, littorine mutase; H6H, hyoscyamine 6β-hydroxylase. The dashed arrow indicates more than one enzymatic step.

Some genera of the family *Solanaceae*, including *Hyoscyamus*, *Duboisia*, *Atropa*, and *Scopolia*, produce biologically active tropane alkaloids (TAs)^1^. TAs are a class of alkaloids characterized by the presence of a bicyclic nitrogen bridge across a seven-carbon ring. The biosynthesis of hyoscyamine and scopolamine is initiated by decarboxylation of the nonproteinogenic amino acid, ornithine^2,3^. The conversion of scopolamine, which is the 6,7-β-epoxide of hyoscyamine, is catalyzed by hyoscyamine 6β-hydroxylase (H6H, EC 1.14.11.11) via two consecutive steps: hydroxylation and epoxidation. In addition, it is known that catalysis of the final reaction in the epoxide formation requires an alkaloid substrate, 2-oxoglutarate, Fe^2+^, molecular oxygen, and ascorbate (Fig. 1)^4,5^.

Among all the TAs, scopolamine has the highest pharmacological efficacy and the least side effects, and is the most important and valuable TA; it is widely used as a mydriatic, antiemetic, antispasmodic, anesthetic, and bronchodilator agents^6^. Owing to these properties, there is an enormous global demand for scopolamine. In recent years, *Hyoscyamus niger* has been shown to be an excellent model plant for determining the efficacy of metabolic engineering approaches aimed at increasing the content of TAs, such as those involving an increase in flux through biosynthetic pathways^1^. In a study published in 2004, the overexpression of *PMT* and *H6H* in transgenic hairy roots of *H*. *niger* was demonstrated to result in a 9-fold greater accumulation of scopolamine than that in the control lines^1^. Similar results were reported for hairy roots of *Atropa belladonna* overexpressing the same genes, wherein the best transgenic line showed 7.3-fold higher content of scopolamine than that in the control line^7^. Although the large-scale culture of genetically engineered *H*. *niger* hairy roots has great potential in terms of the commercial production of TAs, the need for high external critical oxygen tension for cell growth and production of metabolites has remained unaddressed for many years^8,9^.

*Vitreoscilla* soluble hemoglobin (VHb) is characterized as an oxygen carrier and transporter with extraordinarily high k_off_ rate for oxygen release^10,11^. The oxygen-dependent *VHb* promoter was reported to be maximally induced under microaerobic conditions (with dissolved oxygen levels being less than 2% of the air saturation) in its native host^12,13^. The expression of VHb leads to the promotion of host growth and improvement in the production of some metabolic products^14–16^. The functional investigation of VHb suggested that it might increase the activity of terminal oxidases by increasing the local supply of oxygen and by shifting the cellular physiology to the energetically more efficient aerobic state^17–19^.

The beneficial effects of heterologous expression of VHb in plants have been known for more than a decade. *Nicotiana tabacum* plants expressing VHb were reported to grow faster and had a shorter lifecycle than those of the control plants. A shift in secondary metabolite synthesis from anabasine to nicotine, the synthesis of which is more O_2_-demanding, was also observed in the transgenic tobacco plants^20^. The VHb-producing cabbage seeds germinated more rapidly than those of the control plants^21^. Previously, we conducted similar studies employing the expression of VHb for the optimization of plant metabolism. The *VHb* gene was first introduced into *Arabidopsis thaliana* to investigate its positive effects on the metabolism in *Arabidopsis*. The constitutive expression of VHb improved the germination rate of transgenic seeds and affected the levels of some endogenous genes of *Arabidopsis* that are involved in oxygen metabolism and biosynthesis of antioxidants. Moreover, the VHb transgenic plants were more tolerant to photo-oxidative damage because of the increased production of antioxidants, such as ascorbate^16^. These results suggested that the *VHb* gene might potentially be used in molecular breeding for improving the production of TAs in *H*. *niger* hairy roots.

The active regulation of VHb within the metabolic network in each cell is also determined by the enzymes expressed in the cell. For example, localization of VHb to different cellular compartments (cytoplasm, chloroplast, and mitochondria) may result in phenotypic differences in the host^22,23^. Moreover, recent evidence has revealed that VHb is localized to the cytoplasm and is concentrated near the cytosolic face of the cell membrane^24^. Compartmentalization engineering is a promising strategy to increase the productivity of a desired secondary metabolite in plants^25^; for example, *Kappers et al*. introduced a linalool/nerolidol synthase into the mitochondria of *Arabidopsis* by fusing a bona fide mitochondrial targeting signal-CoxIV (cytochrome oxidase subunit IV) sequence from yeast. They reported that mitochondrial targeting of the sesquiterpene synthase resulted in the emission of two new isoprenoids from the *A*. *thaliana* plants and aided in their defense mechanism^26^. This prompted us to investigate the consequences of subcellular targeting of VHb on the production of TAs in the hairy roots of *H*. *niger*.

In this study, we targeted the expression of VHb in the mitochondria and plastids of the hairy roots of *H*. *niger* with the aim of enhancing the production of TAs. The effects of VHb expression on the accumulation of hyoscyamine and scopolamine and on the transcription of TA biosynthesis genes were also investigated. Our results demonstrate that targeted VHb expression significantly increased the TA content in the *H*. *niger* hairy roots, and resulted in the up-regulation of key genes involved in the TA biosynthetic pathway. Overall our results prove that targeted expression of VHb is a simple and efficient approach to increase the production of TAs in the hairy roots of *H*. *niger*.

## Results

### Subcellular localization of VHb expression

**Figure 2:**
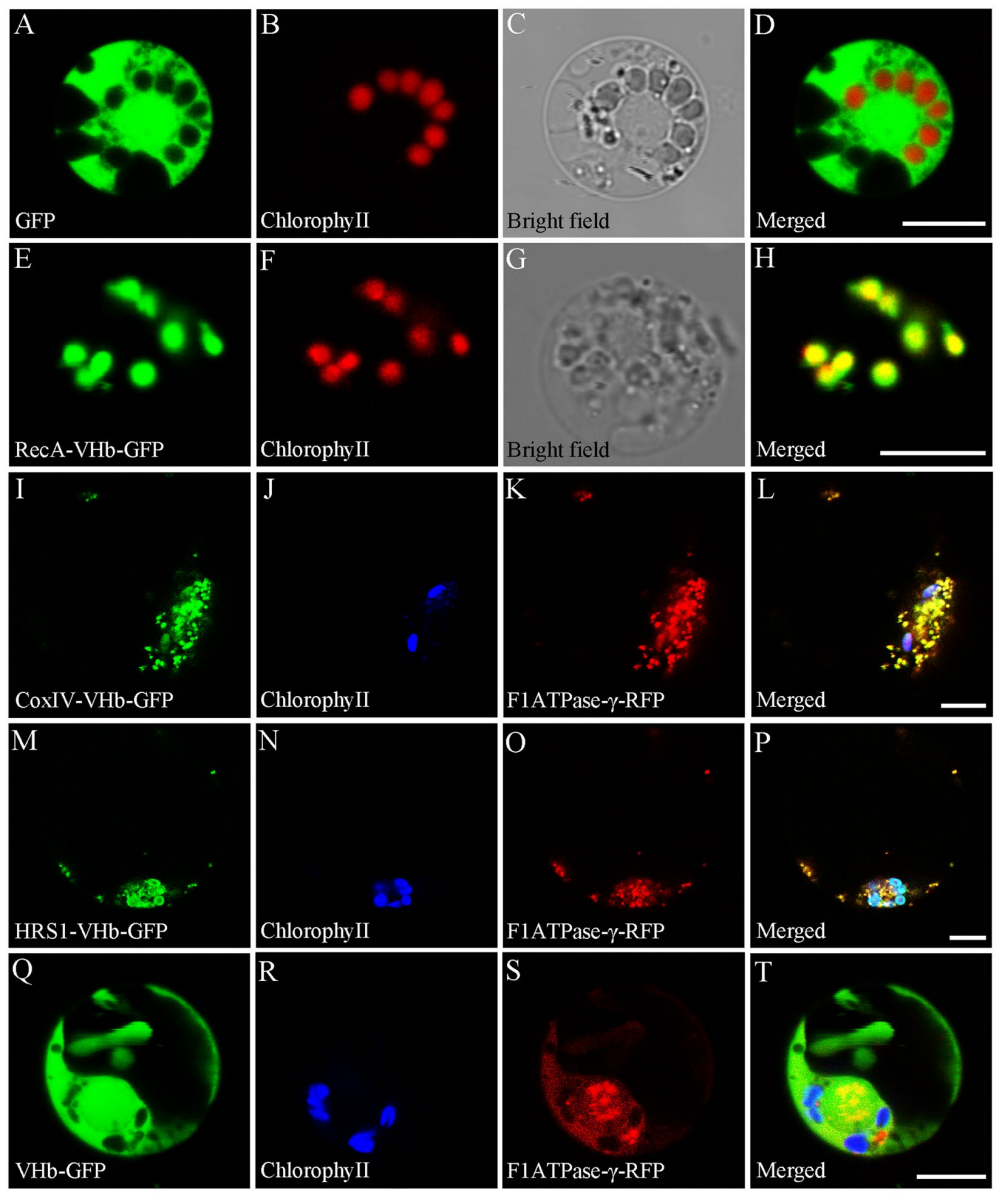
Subcellular localization of VHb, RecA, CoxIV, and AtHRS1. (**A**) A control protoplast expressing free GFP. (**B**) The same protoplast as in (A) showing chlorophyll autofluorescence in the plastids. (**C**) The same protoplast as in (**A**) under white light. (**D**) A merge of images shown in (**A**,**B**). (**E**) Green fluorescence from the chloroplasts of a protoplast expressing *p*VHb-RecA. Protoplasts that expressed free GFP emitted green fluorescence signals similar to that in the controls. (**F**) The same protoplast as in (**E**) showing red fluorescence. (**G**) The same protoplast as in (**E**) under white light. (**H**) A merge of images shown in (**E**,**F**). (I) Green fluorescence from the mitochondria of a protoplast expressing *p*VHb-CoxIV. (**J**) The same protoplast as in (**I**) showing chlorophyll autofluorescence in the plastids. (**K**) The same protoplast as in (**I**) expressing a mitochondrion-localizing F1ATPase-γ-RFP giving a red fluorescence signal. (**L**) A merge of images from (**I**) to (**K**). (**M**) Green fluorescence from the chloroplasts and mitochondria of a protoplast expressing *p*VHb-AtHRS1. (**N**) The same protoplast as in (**M**) showing chlorophyll autofluorescence in the plastids. (**O**) The same protoplast as in (**M**) expressing a mitochondrion-localizing F1ATPase-γ-RFP giving a red fluorescence signal. (**P**) A merge of images from (**M**–**O**). (**Q**) Green fluorescence from the cytoplasm of a protoplast expressing *p*VHb. (**R**) The same protoplast as in (**Q**) showing chlorophyll autofluorescence in the plastids. (**S**) The same protoplast as in (R) expressing a mitochondrion-localizing F1ATPase-γ-RFP giving a red fluorescence signal. (**T**) A merge of images from (**Q**–**S**). Bars = 7.5 μm.

For targeting of VHb to the mitochondria and plastids of *H*. *niger*, a 20-amino acid (AA) N-terminal mitochondrial localization signal from subunit IV of yeast CoxIV, an N-terminal extension with features characteristic of mitochondrial and chloroplast transit peptides from *Arabidopsis* (AtHRS1)^27^, and an *A*. *thaliana* gene that encodes a Mg^2+^- and ATP-dependent RecA homologue targeted to plastids^28^, were selected, respectively. To experimentally verify the subcellular localization of VHb, RecA, CoxIV, and AtHRS1, four binary plant expression fusion constructs, namely *p*VHb, *p*VHb-CoxIV, *p*VHb-RecA, and *p*VHb-AtHRS1 were generated in the *p*CAMBIA1301-GFP background. The *p*CAMBIA1301-GFP construct served as a control. Plastid signals were observed by the autofluorescence of chlorophyll. To mark the mitochondria, a translational fusion construct of the mitochondrial F1ATPase γ-subunit and the red fluorescent protein (RFP)^29^ was co-transformed into the protoplasts. As shown in Fig. 2A–D, free GFP was detected in the cytoplasm. Our analysis revealed that the RecA-VHb-GFP signal co-localized with the chlorophyll autofluorescence signal in the plastids (Fig. 2E–H), suggesting that *p*VHb-RecA was exclusively localized to the plastids. However, the CoxIV-VHb-GFP signal co-localized with the F1ATPase-γ-RFP signal but not obviously with the chlorophyll autofluorescence signal (Fig. 2I–L), indicating that *p*VHb-CoxIV was mainly localized in mitochondria. In addition, the AtHRS1-VHb-GFP signal colocalized with the chlorophyll autofluorescence signal and the F1ATPase-γ-RFP signal (Fig. 2M–P), suggesting that *p*AtHRS1-VHb was localized to the plastids and mitochondria. In contrast, the VHb-GFP signal was similar to that of the free GFP and was found in the cytoplasm and did not colocalize with the RFP signal or the chlorophyll autofluorescence signal (Fig. 2Q–T).

### Gene transformation and confirmation of transgenic lines

**Figure 3:**
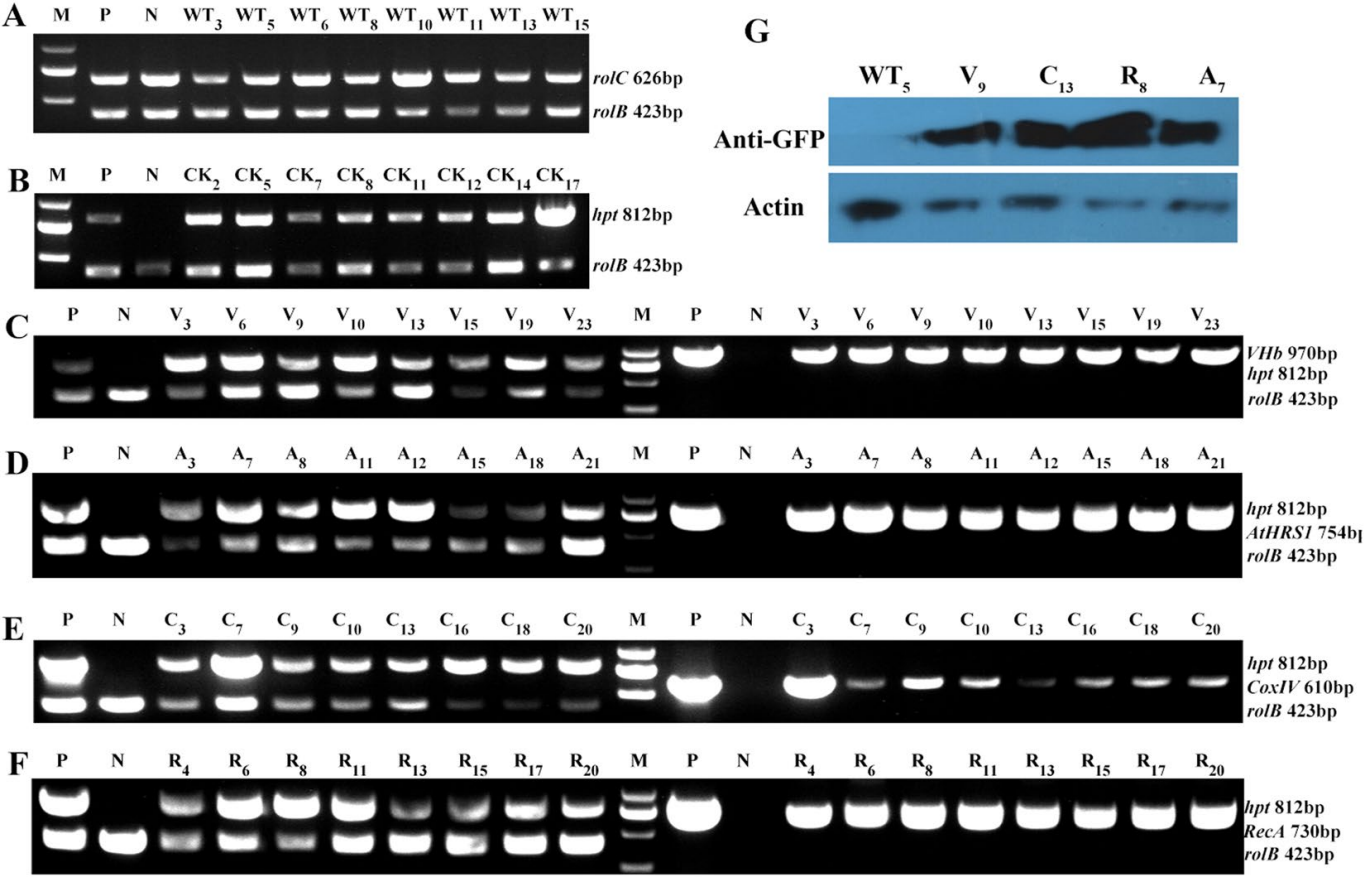
Molecular and western blot analyses for confirmation of transformation of hairy root lines. (**A**) WT hairy roots infected with *Agrobacterium rhizogenes* C58C1. Transgenic hairy root lines infected with *A*. *rhizogenes* C58C1 harboring the binary vector *p*CAMBIA1301-GFP (**B**), *p*VHb (**C**), *p*VHb-AtHRS1 (**D**), *p*VHb-CoxIV (**E**), and *p*VHb-RecA (**F**). The numbers indicate the hairy root lines. (**M**) Molecular size marker (2 kb ladder). (**P**) The corresponding engineered bacteria (positive control). (**N**) WT hairy root (negative control). (**G**) Results of western blot analysis showing the expression of VHb, detected using a GFP-specific monoclonal antibody in non-transgenic and four independent transgenic hairy root lines.

The verified vector constructs were separately introduced into *H*. *niger* leaf disc explants via the *Agrobacterium rhizogenes* C58C1 strain; the generated hairy root lines were screened using hygromycin selection. After culturing for 45 days, 25 *p*VHb (V), 22 *p*VHb-AtHRS1 (A), 20 *p*VHb-CoxIV (C), 20 *p*VHb-RecA (R), 17 CK, and 15 WT hairy root lines were harvested, of which 11 V, 9 A, 10 C, 9 R, 11 CK, and 13 WT lines survived the successive subculture process. From these, every eight lines were selected for RNA extraction, metabolite analysis and qRT-PCR analysis (Table 1). To verify the transformation of pRiA4^30^, the *rol* genes were detected via PCR analysis of all the lines. As shown in Fig. 3A–F, none of the analyzed DNA fragments was amplified from the WT root samples. To further verify the expression of VHb, the expression of GFP was detected by western blot analysis using a GFP-specific monoclonal antibody (Fig. 3G). The above results confirmed that *VHb* and subcellular localization sequences had successfully integrated into the genome of *H*. *niger* hairy roots (Fig. 3). Overall, 82.05% (32/39) of the generated hairy root lines were PCR-positive and hygromycin tolerant; the break-up of this figure for individual constructs was 88.89% (8/9) for A, 80% (8/10) for C, 88.89% (8/9) for R, and 72.72% (8/11) for V.

**Table 1:** Gene constructs and derived root cultures.

T-DNA construct	Number of established root lines			Established root cultures
	total	Antibiotic-resistant	PCR-positive	
<AtHRS1 < VHb < hpt	22	9	8	A_3_, A_7_, A_8_, A_11_, A_12_, A_15_, A_18_, A_21_
<CoxIV < VHb < hpt	20	10	8	C_3_, C_7_, C_9_, C_10_, C_13_, C_16_, C_18_, C_20_
<RecA < VHb < hpt	20	9	8	R_4_, R_6_, R_8_, R_11_, R_13_, R_15_, R_17_, R_20_
<VHb < hpt	25	11	8	V_3_, V_6_, V_9_, V_10_, V_13_, V_15_, V_19_, V_20_

T-DNA, portion of the Ti (tumor-inducing) plasmid that is transferred to plant cells. The antibiotic resistance gene (*hpt*) is always placed near the left border of the T-DNA. In addition, seven transformed with C58C1 were also established, as WT control.

### Growth characteristics of transgenic lines

**Figure 4:**
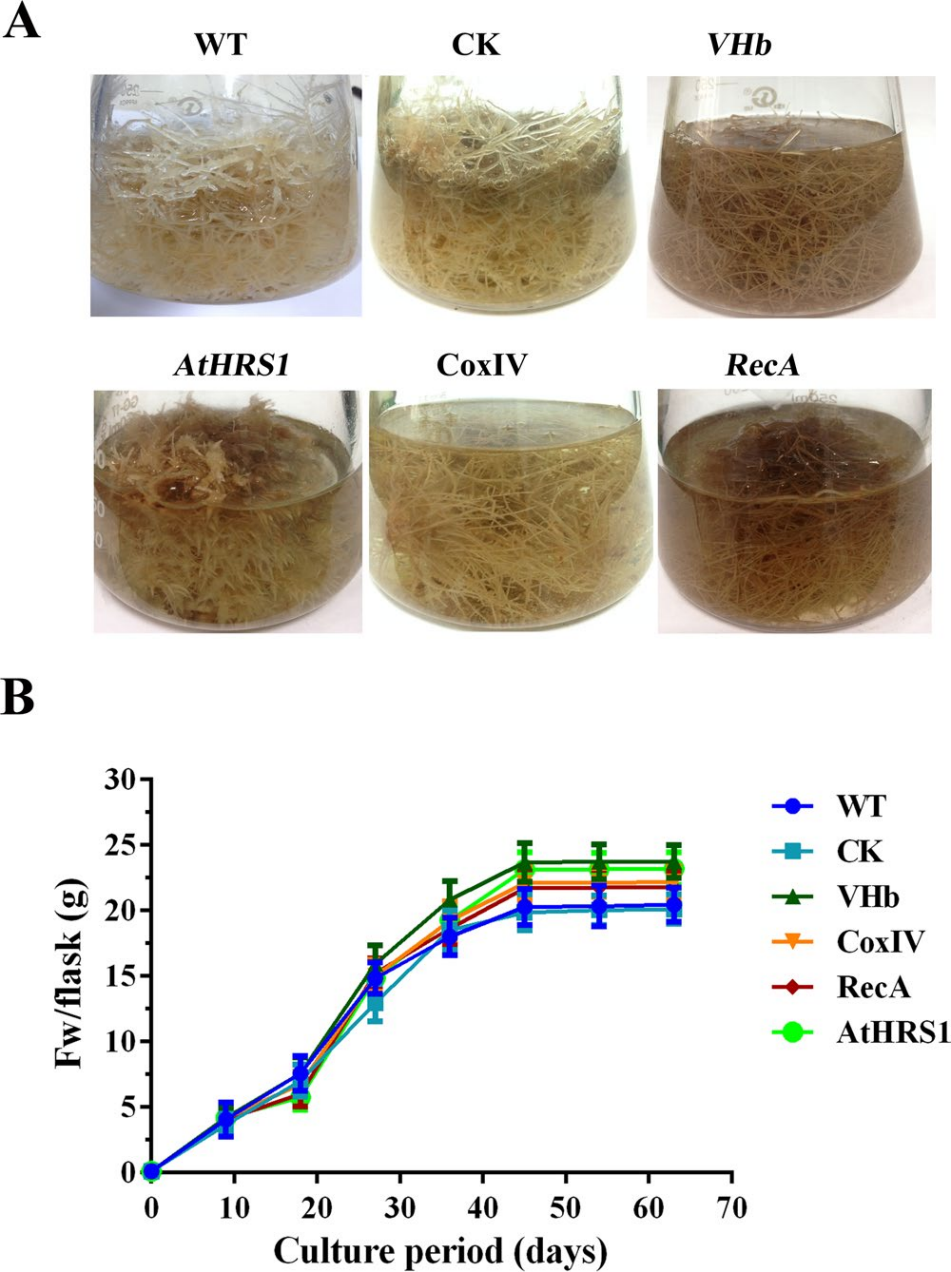
Analyses of the morphology and growth rate in WT and transgenic hairy root lines. (**A**) Phenotype of root lines developed in liquid 1/2 MS medium for 45 days. (**B**) Time course of growth of transgenic hairy root lines. Each set of lines corresponds to one transgenic line and within each set, the replicates of independent transgenic lines are as depicted in Table 1. Error bars show the standard deviations for the independent lines.

The morphology and growth rate of the hairy root lines were evaluated by determining the increase in fresh weight (FW). WT, VHb-expressing, and CK lines showed a similar growth curve (Fig. 4A). In shake-flask cultures, the biomass of *H*. *niger* hairy roots exhibited a steady, linearly increasing trend during the growth period. All the WT, CK, and transgenic lines grew rapidly and vigorously and had thick branches. Whereas the biomass of WT lines increased more rapidly during the first nine days, that of VHb transgenic lines showed a significant increase from the 9th day (i.e., during the logarithmic phase). All the hairy root lines entered a stagnant phase after 45 days (Fig. 4B). The difference in the growth was not significant (*p* > 0.05) among the WT, CK, and *p*VHb, 22 *p*VHb-AtHRS1, 20 *p*VHb-CoxIV, and 20 *p*VHb-RecA transgenic lines.

### Targeted expression of VHb promotes higher accumulation of tropane alkaloids in *H*. *niger* hairy roots

**Figure 5:**
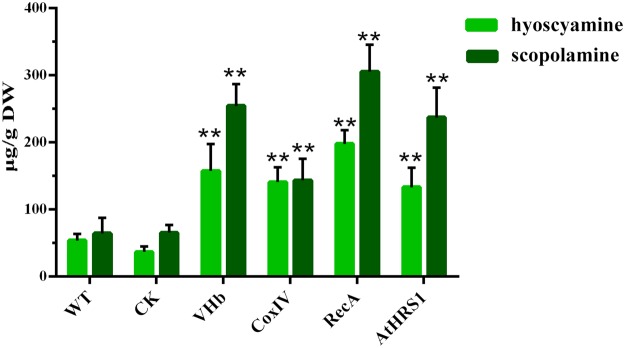
Total content (µg/g DW) of tropane alkaloids (hyoscyamine and scopolamine) in different hairy root lines at the end of the culture period (45 days). The concentrations of the alkaloids were determined using LC-MS/MS. Each set of bars corresponds to one transgenic line, and within each set, the replicates of independent transgenic lines are as depicted in Table 1. Error bars show the standard deviations for the independent lines. The level of significance obtained using One-Way ANOVA is indicated as follows: **p < 0.01.

The contents of hyoscyamine and scopolamine in the WT, CK, and transgenic hairy root lines were analyzed using liquid chromatography-tandem mass spectrometry (LC-MS/MS). The production of TAs could be detected in both the transgenic and non-transgenic hairy roots but varied in the different lines (Fig. 5). Interestingly, the production of TAs was considerably lower in the *p*CAMBIA1301-GFP transgenic lines than in WT. The accumulation of TAs was stimulated by the expression of VHb in the *H*. *niger* hairy roots and the increase in the hyoscyamine content varied from 2.5- to 3.6-fold in the four transgenic lines with respect to the content in the non-transgenic lines (53.91 μg/g DW) (Fig. 5). Moreover, accurate subcellular localization of VHb increased the accumulation of TAs. The maximum content of hyoscyamine was 197.68 μg/g DW when VHb expression was targeted to the plastids (*p*VHb-RecA); it was 1.25-fold of that in the lines in which VHb was expressed in a non-targeted manner (*p*VHb). The increase in the content of scopolamine varied from 2.2- to 4.7-fold in the four transgenic lines with respect to the content in the non-transgenic lines (64.38 μg/g DW) (Fig. 5). The highest concentration of scopolamine (305.51 μg/g) was found in the *p*VHb-RecA lines, which was 1.20-fold of that in the lines in which VHb expression was not targeted (*p*VHb).

### Expression of tropane alkaloid biosynthesis genes is significant higher in transgenic hairy roots expressing VHb

**Figure 6:**
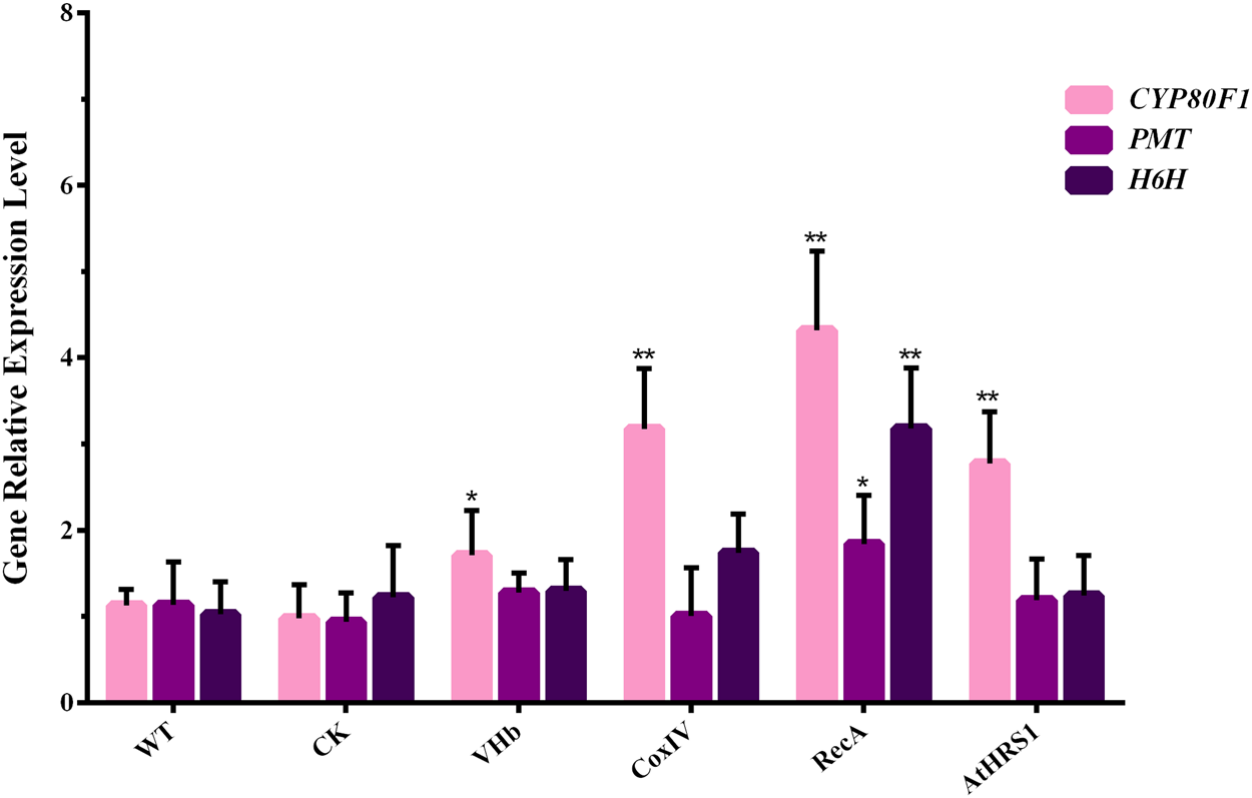
Quantification of the transcripts of genes involved in the biosynthesis of tropane alkaloids in transgenic hairy root lines. Each set of bars corresponds to one transgenic line and, within each set, each color corresponds to a single gene. The replicates of independent transgenic lines are as depicted in Table 1. Error bars show the standard deviations for the independent lines. The level of significance obtained using One-Way ANOVA is indicated as follows: *p < 0.05, **p < 0.01.

**Figure 7:**
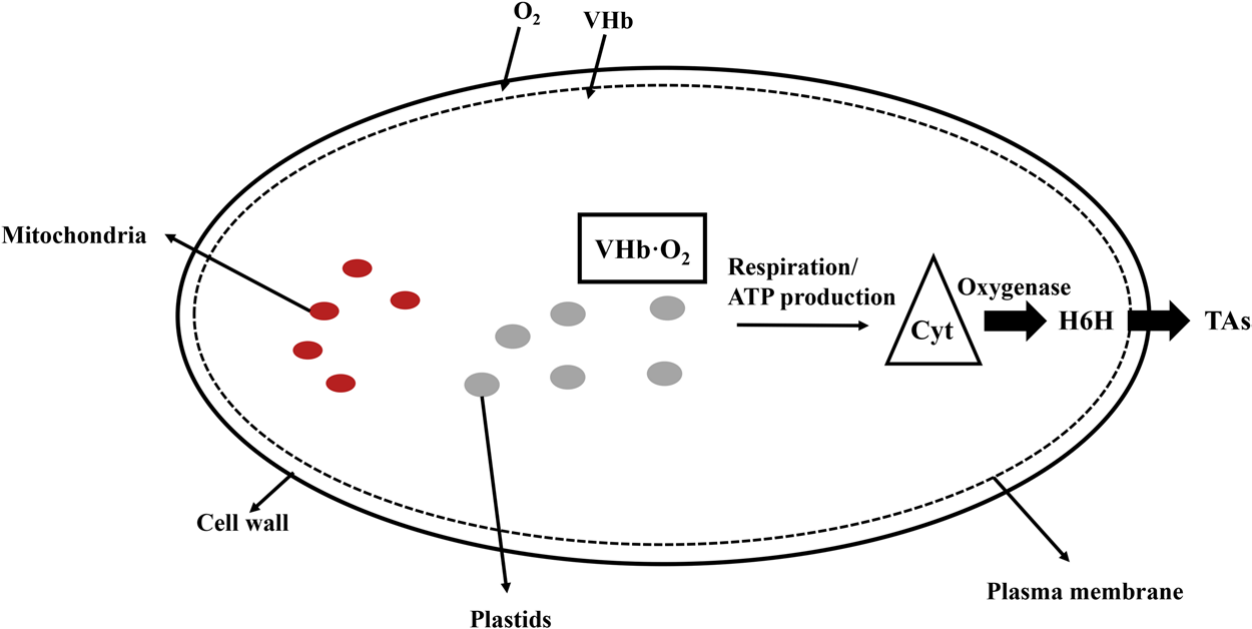
Schematic model of targeted VHb expression in different subcellular compartments regulating the tropane alkaloid biosynthesis. Oxygen enters the cell and binds to VHb (depicted by dots concentrated underneath the plasma membrane). The VHb bound to oxygen interacts with various partner proteins, delivering oxygen to oxygenases that enhances the activities of Cyt and H6H and, thus, increases the accumulation of TAs.

To date, research on TA biosynthesis has focused on *PMT* and *H6H* genes, which encode the rate-limiting enzymes that can be genetically modified in *H*. *niger* plants. Previously, simultaneous introduction and overexpression of genes encoding the rate-limiting upstream enzyme PMT and the downstream enzyme H6H in transgenic *H*. *niger* hairy root cultures was reported^1,7^. We concluded that transgenic plants harboring both *PMT* and *H6H* had an increased flux through the TA biosynthetic pathway that enhanced the yield of scopolamine, which was more efficiently produced than in the plants harboring only one of these genes. Moreover, studies have shown that cytochrome P450 can catalyze a series of oxidation reactions, and the evolution of oxygen occurs mainly in the chloroplasts^31,32^. Therefore, the expression levels of the three genes in the TA biosynthetic pathway, namely *PMT*, *H6H*, and *CYP80F1*, were determined using qRT-PCR to study the effects of VHb on transcription. The transcription levels of *PMT* and *H6H* increased marginally in the *p*VHb-RecA line, whereas in the *p*VHb-CoxIV and *p*VHb-AtHRS1 lines, the expression levels were similar to those in the control lines. In comparison to the CK-transformed control lines, the transcript levels of *CYP80F1* in the *p*VHb, *p*VHb-RecA, *p*VHb-CoxIV, and *p*VHb-AtHRS1 lines were coordinately enhanced (the increase in expression being 1.7- to 4.4-fold, *p < *0.05). The highest *CYP80F1* expression was observed in the *p*VHb-RecA lines, which showed a 4-fold increase compared to the expression levels in the non-transgenic lines, an observation that was consistent with the increase in the content of TAs (Fig. 6). In light of these results, the functions of VHb are summarized in Fig. 7. We speculate that VHb accelerates the biosynthesis of TAs by enhancing the utilization of oxygen and by increasing the flux through the pathway.

## Discussion

The biosynthesis of TAs in various plants has recently drawn considerable attention. *H*. *niger* is one of the most effective plant producers of TAs; these compounds are biosynthesized in the roots^33^. Although the content of TAs in cultured plant cells can be increased to the levels that are obtained from whole plants, the process results in unstable product yield and high production costs^34^. Metabolic engineering of TA biosynthetic pathways, either by overexpression of key genes or by elicitation using different plant hormones in *Hyoscyamus muticus*^35^, *Scopolia parviflora*^36^, and *Duboisia hybrid*^37^, is the approach that is commonly used. However, the engineering of biosynthetic pathways that function in different cellular compartments has largely been ignored. The expression of VHb has been shown to be a promising genetic strategy for combating oxygen limitation and for improving the phenotypic properties of *Vitreoscilla*^38^. Moreover, heterologous expression of VHb was shown to improve the growth of *H*. *muticu*s hairy roots as well as it increased the volumetric production of hyoscyamine in the VHb lines under conditions of limited oxygen availability^39,40^. The presence of VHb causes significant metabolic changes during the aerobic growth of several organisms; however, the mechanism of VHb action remains to be elucidated. In this study, accurate subcellular metabolic engineering was done to engineer the production of TAs in the plastids of *H*. *niger* hairy roots. In addition, the function of VHb was explored at the subcellular level for the first time.

Mitochondria and plastids are the pivotal organelles in plant cells^32^. The ATP synthases present in mitochondria and plastids are key enzymes in plant metabolism, involved in the generation of ATP, the universal energy currency of the cell^41^. The mitochondria contain diverse types of metabolites as well as high levels of cofactors, such as NADH, NAD^+^, FAD, and acetyl-CoA. Many important processes, such as the evolution of oxygen, occur in plastids^32^. All these aspects can be exploited for metabolic engineering of TA with the aid of mitochondrial and/or plastid targeting sequences. Unlike the case of cytoplasmic localization of VHb in the *p*VHb lines, *p*RecA-VHb was faithfully targeted to the plastids, *p*CoxIV-VHb fluorescence was detected in the mitochondria, and *p*AtHRS1-VHb was faithfully targeted both to the plastids and mitochondria.

The integration of the VHb gene into the genome of the hairy roots of *H*. *niger* was confirmed by genomic PCR using VHb-specific primers. Despite the targeting of VHb to different cellular compartments, no significant differences in morphological characteristics were observed among the different lines (Fig. 4A). The FWs of VHb-expressing hairy root cultures were on average 16% higher than those of the controls (Fig. 4B), indicating that the location of the integration site was “neutral” and that VHb improved the growth of *H*. *niger* hairy roots. A relatively high content of secondary metabolites in tissues is usually associated with poor growth and, therefore, the actual total productivity of secondary metabolites remains low^42^. In this study, we found that the growth rate of transgenic lines with high TA production was not reduced when compared with the lines that had low production of TA. This result was similar to that obtained for *H*. *muticus*^39^ and *N*. *tabacum*^22^ but was contrary to those obtained for *Arabidopsis* and maize^43^. The presence of VHb did not cause a significant increase in the growth of hairy roots (Fig. 4B), possibly because of the fact that the native promoter limited the expression of VHb, or because of sufficient supply of oxygen, and a relatively low cell density in the WT, CK, and VHb-expressing lines under normal culture conditions^44^.

TAs are a class of alkaloids defined by the presence of a bicyclic nitrogen bridge across a seven-carbon ring. A series of oxidation reactions catalyzed by cytochrome P450 are involved in the littorine rearrangement that occurs in the TA biosynthesis. Previous reports showed that VHb could stimulate oxygenase activity via direct delivery of oxygen^23,45^. Our results show that VHb expression enhanced the accumulation of hyoscyamine and scopolamine in the hairy roots of *H*. *niger* (Fig. 5), which could enhance the utilization of oxygen and, thereby, accelerate the oxygen-dependent steps in the TA biosynthesis pathway. The targeted expression of VHb to different subcellular compartments increased the transcript levels of *CYP80F1*, *PMT*, and *H6H*, especially in the plastids (Fig. 6) and increased the metabolic flux toward the biosynthesis of scopolamine (Fig. 7).

In summary, heterologous VHb was expressed in the hairy roots of *H*. *niger* under the control of the cauliflower mosaic virus (CaMV) 35S promoter. The expression of VHb in the plastids significantly enhanced the yield of TAs in comparison to their content in transgenic lines expressing this gene in a non-targeted manner and in the control lines. Our research, evaluating the expression of TA pathway genes and secondary metabolism in genetically modified lines, could lead to a more in-depth understanding of the function of heterologous VHb.

## Materials and Methods

### Plant materials

*H*. *niger* seeds were obtained from the Plant Garden of Shenyang Pharmaceutical University, Shenyang, China. To obtain sterilized seedlings, the *H*. *niger* seeds were surface-sterilized in 75% ethanol for 3 min, washed five times with distilled water, and then treated with sodium hypochlorite solution, containing 33% active chlorine, for 4 min. After thorough rinsing five times with sterile distilled water, the sterilized seeds were incubated between several layers of sterilized wet filter paper and placed on MS basal medium (Sigma, USA), the pH of which was adjusted to 5.8–6.0, for germination. The culture room was maintained at a temperature of 25 °C; the light intensity was 350 μmol. m^−2^ s^−1^, provided by white fluorescent tubes in 16-h photoperiods, and the relative air humidity was maintained at 30%. Sterile plants of *H*. *niger* were prepared as described above and 2-month-old seedlings were used for the *Agrobacterium*-mediated transformation experiments.

### Construction of binary plant expression vectors for subcellular targeting of VHb

**Figure 8:**
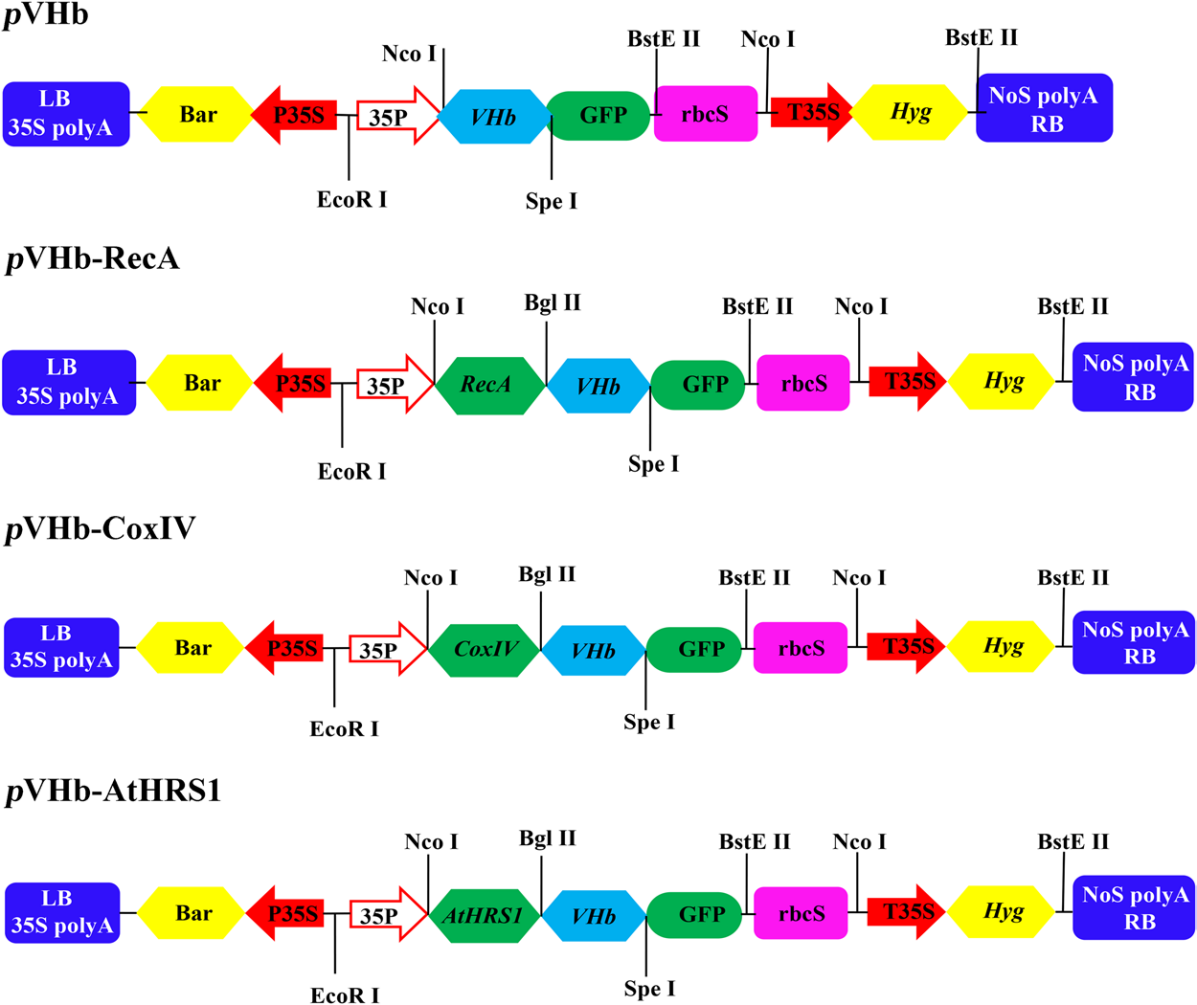
Schematic representation of subcellular localization plasmids used in transformation. P35S, CaMV 35 S promoter; Hyg, hygromycin-resistance gene; GFP, green fluorescent protein coding sequence; T35S, CaMV 35S terminator; LB, T-DNA left border; RB, T-DNA right border. Restriction sites and target genes are indicated.

A 441-bp fragment containing the complete coding sequence of the VHb gene (GenBank accession no. M30794) and a 233-bp fragment of the AtHRS1 gene, containing the localization sequences with *Nco*I and *Bgl*II restriction sites, were synthesized by GENWIZ Biotechnology (Co., Ltd., Suzhou, China). The addition of the remaining two localization sequences was done as follow: *Arabidopsis RecA* and yeast *CoxIV* were PCR-amplified (using the primers shown in Supplementary Table S1) from cDNA and *Saccharomyces cerevisiae* genomic DNA, respectively, as described by *Akashi*^27^. The GUS DNA fragment in the *p*CAMBIA1301 vector was changed to GFP under the control of the CaMV 35 S promoter, generating the modified *p*CAMBIA1301-GFP vector for use in subcellular localization. The *VHb* gene was excised with *Bgl*II and *Spe*I and subsequently inserted into the *p*CAMBIA1301-GFP vector, generating *p*CAMBIA1301-GFP-VHb (*p*VHb). For the *p*CAMBIA1301-GFP-VHb-AtHRS1 (*p*VHb-AtHRS1), *p*CAMBIA1301-GFP-VHb-RecA (*p*VHb-RecA), and *p*CAMBIA1301-GFP-VHb-CoxIV (*p*VHb-CoxIV) binary expression vectors, the *AtHRS*1, *RecA*, and *CoxIV* fragments were subcloned into the *p*VHb vector, generating plasmids *p*VHb-AtHRS1, *p*VHb-RecA, and *p*VHb-CoxIV, respectively (Fig. 8). The clones were verified by PCR and DNA sequencing.

### Analysis of subcellular localization of *p*VHb, *p*VHb-CoxIV, *p*VHb-RecA, and *p*VHb-AtHRS1 fusion proteins

The *p*VHb, *p*VHb-CoxIV, *p*VHb-RecA, and *p*VHb-AtHRS1 vectors that contained the GFP tag were transformed into freshly prepared *Oryza sativa* protoplasts using PEG-mediated transformation; this was followed by transformation with the control mitochondrial F1ATPase-g-RFP fusion construct (kindly provided by Hyun-Sook Paic, Yonsei University, Korea)^29^.The transfected protoplasts were incubated overnight at room temperature and transient expression was visualized using a confocal laser scanning microscope (Nikon, Japan) as described by Tan^46^. The GFP fluorescence was visualized using an excitation wavelength of 488 nm and emission wavelengths in the range from 505 to 530 nm. The red autofluorescence of chlorophyll was captured at an emission wavelength longer than 650 nm. The captured images were processed using Photoshop software.

### Plant transformation and hairy root culture

The transformation of leaf explants from the *H*. *niger* plants was carried out following a previously described method^1^, and simultaneous transformation with C58C1 strains was done to serve as the wild-type control^47^. The culture of *H*. *niger* hairy roots was obtained after the infection of leaf explants with C58C1 and these roots were developed at the cut edges after 1–2 week of co-cultivation. They were then excised and cultured on solid, hormone-free, half-strength MS medium supplemented with 30 g/L sucrose, which served as the carbon source^42^. All the culture media contained 100 mg/L hygromycin and 500 mg/L cefotaxime. The rapidly growing hygromycin-resistant lines, without bacterial contamination, were used to establish the hairy root lines. Approximately 100 mg of fresh roots were inoculated into 250 mL conical flasks containing 200 mL of liquid half-strength MS medium, cultured on an orbital shaker (110 rpm) at 25 °C in the dark, and the culture medium was refreshed every nine days. The FW of root tissues from the culture flasks was recorded individually after 9, 18, 27, 36, and 45 days of inoculation. At day 45, the harvested hairy roots (0.1 g) were used for DNA and RNA isolation; the remaining hairy roots were separated from the culture medium by filtration, washed three times with distilled water, blotted dry on paper towels to yield the FW, and then dried at 45 °C in an oven until a constant DW was obtained. The experiment was performed in triplicate, and the results presented are means ± S.E.M.

### Analysis of the transformed lines by PCR

The presence of the transformed *p*VHb, *p*VHb-AtHRS1, *p*VHb-RecA, and *p*VHb-CoxIV genes was examined using PCR with the primers shown in Supplementary Table S1. Genomic DNA was isolated from hairy root samples using a Qiagen DNeasy Plant Mini Kit (Qiagen Biotech, China). The DNA was then used to detect the presence of the specific genes in the transgenic lines by PCR. To detect the presence of exogenous genes in the transgenic lines, the primer 35S-F and the reverse primers for *VHb*, *AtHRS1*, *CoxIV*, and *RecA* were specially designed so as to cover both the gene and the vector sequences. The selectable marker gene, *hygromycin phosphotransferase* (*hpt)*, was used to confirm the empty vector transformants, whereas the *Agrobacterium rolB* and *rolC* genes were used to confirm the transformation of pRiA4^30^.

### Western blot analysis

The fresh hairy root samples were homogenized in 500 μL of plant protein extraction reagent containing 1X protease inhibitor cocktail, according to the manufacturer’s recommendation (CoWin Biosciences, China). The whole protein extracts from the *H*. *niger* transgenic lines and control lines were mixed with 5X sample loading buffer (50% glycerol, 10% SDS, 5% β-mercaptoethanol, 0.5% bromophenol blue, and 0.25 M Tris, pH 6.8), heated to 100 °C for 5 min, loaded onto a 10% SDS-polyacrylamide gel, and electrophoresed at 80 V for 120 min in a Bio-Rad Mini Trans-Blot cell. The total protein was subsequently transferred to PVDF membrane. The membranes were blocked using 5% non-fat dry milk in PBS-0.05% Tween-20 and incubated overnight with the primary antibody (anti-β-actin, Sigma, USA or anti-GFP, Takara, Japan) at a 1:5000 dilution in 3% Bovine serum albumin. The blots were washed three times with TBST (137 mM NaCl, 2.7 mM KCl, 10 mM Na_2_HPO_4_, 2 mM KH_2_PO_4_, 0.3% Tween-20 (V/V)) and incubated for 4 h with goat anti-mouse IgG antibody (1:5000) (Abmart, China). The bands were developed using a chemiluminescent substrate (Thermo, USA).

### Extraction and LC-MS/MS analysis of tropane alkaloids in transgenic hairy roots

The extraction of tropane alkaloids (hyoscyamine and scopolamine) was based essentially on the method described by *Kamada et al*.^48^, with some modifications. The hairy roots were collected and dried at 45 °C in an oven until a constant DW was attained. They were then powdered finely and 50 mg of the powdered sample was sonicated in polypropylene test tubes with an extraction solvent consisting of methanol:ammonia (1:1, v/v) (4 mL) for 45 min; when fully oscillated, 6 mL chloroform was added and the mixture was kept at room temperature overnight. After freeze-drying, the residue was dissolved in 2 mL of methanol (sonicated for 3 min, if insoluble substances were present), and then centrifuged at 10,000 × g for 5 min. The supernatant was diluted ten times with methanol, and the extract was filtered through a 0.2-μm filter membrane before analysis.

The contents of hyoscyamine and scopolamine were determined by LC-MS/MS. The analysis was performed on an Agilent 1200 infinity LC column coupled with an Agilent 6410 Triple Quadrupole LC/MS System (Agilent, USA). The LC operating conditions were as follows: LC column, Agilent ZORBAX SB-C18, 2.1 × 100 mm, 3.5 μm silica; mobile phase: acetonitrile/0.051% formic acid with 5 mM sodium acetate (32:68); total flow rate of mobile phase, 0.3 mL/min; run time, 1.8 min. The injection volume was 2 μL. The MS/MS was operated with an electrospray ionization source in positive ion mode. The pressure of the nebulizer gas was set at 40 psi, the source temperature was 350 °C, and the flow rate of gas was 10 L/min. The capillary voltage was 4000 V (positive mode). High-purity nitrogen gas was used as the collision cell gas. The raw chromatograph and the mass spectrogram data were processed with the Mass Hunter Workstation software (Agilent, USA). Multiple reaction monitoring mode (MRM) was used for quantification, and the selected transitions of m/z were 290.3R124.2 for hyoscyamine and 304.21R138.2 for scopolamine (Supplementary Fig. S1). All the standards were purchased from Sigma-Aldrich (St. Louis, MO). The quantification ranges of hyoscyamine and scopolamine were linear, from 1 to 5000 ng/mL (R^2^ = 0.9993) and from 1 to 3000 ng/mL (R^2^ = 0.9996), respectively.

### Real-time quantitative PCR (qRT-PCR)

The qRT-PCR analysis was performed to determine the transcript abundance of *VHb* and other synthetic genes of the tropane alkaloid biosynthesis pathway, including *CYP80F1*, *H6H*, and *PMT* in the WT, CK, and transgenic hairy roots of *H*. *niger*. Total RNA was extracted from *H*. *niger* hairy roots using TRIzol A^+^ Reagent (Tiangen Biotech, China). The quality and concentration of RNA were examined by NanoDrop (Thermo, USA) and by visualizing the bands on ethidium bromide-stained agarose gels. The total RNA (1 μg) was reverse-transcribed to obtain cDNA using TransScript First-Strand cDNA Synthesis SuperMix Kit (TaKaRa, Japan), according to the manufacturer’s instructions. qRT-PCR was performed following the instructions from the SYBR-Green PCR Master Mix Kit (Takara, Japan) on a Thermal Cycler Dice Real Time System TP800 (Takara, Japan). An efficiency-corrected comparative Ct method^49^ was applied and the relative expression of genes was calculated by normalizing the expression of the genes of interest to the abundance of the housekeeping gene (*18S* ribosomal subunit). All the qRT-PCR experiments were performed in three independent replicates. All the primers used for qRT-PCR are shown in Supplementary Table S1.

### Statistical analysis

The experiments were performed in triplicate, and all the results are expressed as means ± SEM. All statistical analysis was performed using One-Way ANOVA., which was followed by Tukey’s pairwise comparison test, at a level of *p* < 0.05, to determine significant differences between the means.

## Electronic supplementary material

supplementary information
