# Supplementary material for: Targeted expression of Vitreoscilla hemoglobin improves the production of tropane alkaloids in Hyoscyamus niger hairy roots
Source: Sci Rep. 2018 Dec 19;8:17969. doi: 10.1038/s41598-018-36156-y (PMC6299274; doi:10.1038/s41598-018-36156-y)
Supplement: Supplementary file 1 — supplementary information [file 41598_2018_36156_MOESM1_ESM.pdf]

Targeted expression of *Vitreoscilla* hemoglobin improves the production of tropane alkaloids in *Hyoscyamus niger* hairy roots

Zhiying Guo[1,2,3*], Hexin Tan[3*], Zongyou Lv[3], Qian Ji[3], Yuxiang Huang[1], Jingjing Liu[2], Donghong Chen[2], Yong Diao[1], Jinping Si[2] and Lei Zhang[2,3]

1 School of Biomedical sciences, Huaqiao University, Quanzhou, Fujian 362000, China

2 State Key Laboratory of Subtropical Silviculture, Zhejiang A & F University, Hangzhou, Zhejiang 311300, China.

3 Department of Pharmaceutical Botany, School of Pharmacy, Naval Medical University (Second Military Medical University), Shanghai 200433, China

[*]These authors contributed equally to this work. Correspondence and requests for materials should be addressed to L.Z. (email: zhanglei@smmu.edu.cn or leizhang100@163.com)

Supplementary Table S1. PCR primers used in the text.

Primers used for PCR analysis			
rimer names	***Purpose***	***Primer Sequences (5'-3')***	**Base Pai**
ubVHb-F-*Bgl*II	For subcellular localization	AAAGATCTATGTTAGACCAGCAAACCAT	28
ubVHb-R-*Spe*I	For subcellular localization	AAACTAGTTTCAACCGCTTGAGCGTACA	28
ubCoxIV-F-*Nco*I	For subcellular localization	AGCACCATGGTTTCACTACGTCAATCTA	28
ubCoxIV-R-*Bgl*II	For subcellular localization	AAAGATCTTTTTTGCTGAAGCAG	23
ubRecA-F-*Nco*I	For subcellular localization	AGAACCATGGATTCACAGCTAGTCTT	26
ubRecA-R-*Bgl*II	For subcellular localization	AAAGATCTTCTGTCATCGAATTCAG	25
ubAtHRS1-F-*Nco*I	For subcellular localization	CACTCCATGGTCGACCCACGCGTCC	25
ubAtHRS1-R-*Bgl*II	For subcellular localization	AAAGATCTGATTGACATCGATCTTCTT	27
lb-F	For molecular analysis	TCGCCATGCCTCACCAACTCAC	22
lb-R	For molecular analysis	CCTTGATCGAGCCGGGTGAGAA	22
lc-F	For molecular analysis	CGAGGGGATCCGATTTGCTT	20
lc-R	For molecular analysis	GACGCCCTCCTCGCCTTCCT	20
t-F	For molecular analysis	CGATTTGTGTACGCCCGACAGTC	23
t-R	For molecular analysis	CGATGTAGGAGGGCGTGGATATG	23
5S-F	For molecular analysis	GAGGACCTAACAGAACTCGCC	21
VHb-F	For qRT-PCR	GCCAAACACCCTGAAGTACG	20
VHb-R	For qRT-PCR	ACGCCTGCTTGACAATGTTT	20
18S-F	For qRT-PCR	AAACGGCTACCACATCCAAG	20
18S-R	For qRT-PCR	CCTCCAATGGATCCTCGTTA	20
YP80F1-F	For qRT-PCR	TGTGTGGAAAATGGCAAGGG	20
YP80F1-R	For qRT-PCR	GCCAAAGATTGTCCAGCACA	20
PMT-F	For qRT-PCR	CTACCTAGCAGCAGGATTCG	20
PMT-R	For qRT-PCR	GGTCAGAAGAGTCCACAATAATG	23
H6H-F	For qRT-PCR	GCCCAGACCCAAGTTCAAC	19
H6H-R	For qRT-PCR	CAGCAATCCAGGTAGCATCC	20

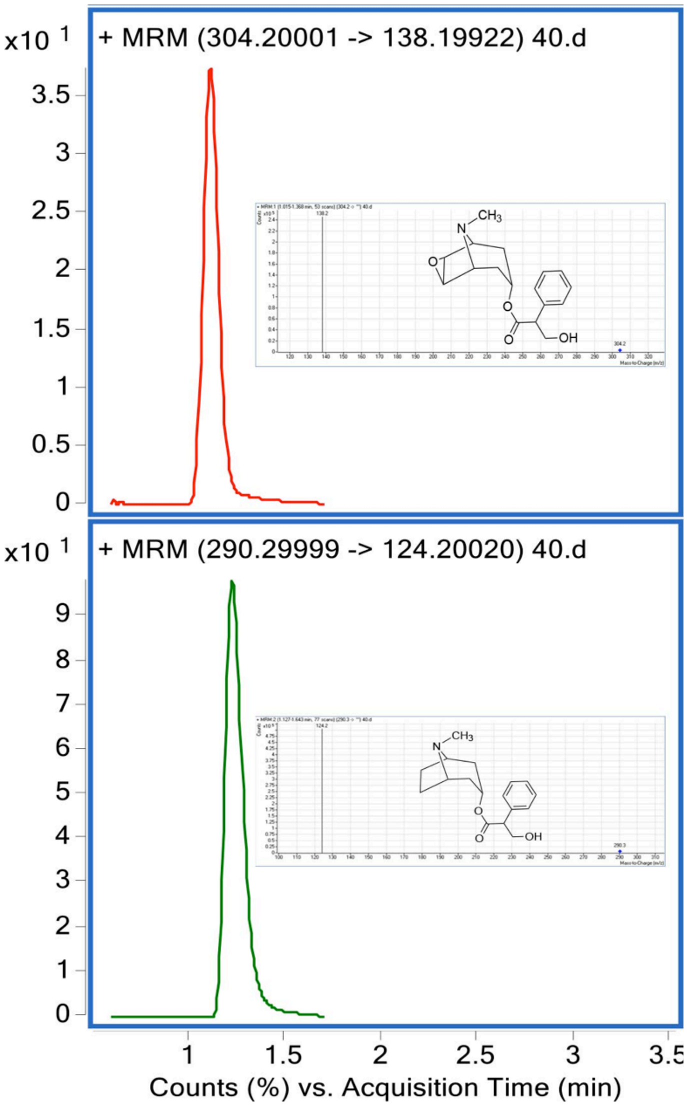

Supplementary Figure S1. Chromatogram result of VHb-expression transgenic hairy roots by electrospray LC-MS/MS. The hyoscyamine and scopolamine compounds were detected using ESI in negative-ion mode, quantified by multiple-reaction monitoring (MRM) mode using following transition mass of m/z, 290.3→124.2 for hyoscyamine, 304.2→138.2 for scopolamine. The extracted peak identification: (A) hyoscyamine (290) and (B) scopolamine (304). This chromatogram is the result of V1 transgenic line. All standards were purchased from Sigma-Aldrich (St. Louis, MO). Data analysis was carried out using the Agilent Mass Hunter Workstation software.
